# Supplementary material for: Evaluation of Two Vaccines against Foot-and-Mouth Disease Used in Transcaucasian Countries by Small-Scale Immunogenicity Studies Conducted in Georgia, Azerbaijan and Armenia
Source: Vaccines (Basel). 2024 Mar 12;12(3):295. doi: 10.3390/vaccines12030295 (PMC10975580; doi:10.3390/vaccines12030295)
Supplement: Supplementary file 1 [file vaccines-12-00295-s001.zip › vaccines-2874435-supplementary.pdf]

vaccines

Supplementary Figure S1. Percentage of Large Ruminants (LR) immunoreactive against FMDV serotype O. Graphs showing the percentage of LR sera with positive VNT titres ($log_{10} \geq 1.5$) against FMDV strain O/ME-SA/PanAsia-2/TUR/07. Sera from Georgia (a), Azerbaijan (b) and Armenia (c). Abscissa, days post vaccination (DPV); ordinate, percentage of animals with log_{10} VNT titres crossing the positivity threshold (>1.5). Empty bars, single vaccinated animals (LR-01 and LR-02 before booster); filled bars, double vaccinated animals (LR-02 group after booster). #, the positivity percentage was not indicated because the group is too small (1 or 2 animals).

Supplementary Figure S2. Percentage of Large Ruminants (LR) immunoreactive against FMDV serotype A. Graphs showing the percentage of LR sera with positive VNT titres ($log_{10} \geq 1.5$) against FMDV strain A/ASIA/Iran-05/TUR/06 (a-c) and FMDV strain A/ASIA/G-VII/NEP/84 (d-f). Sera from Georgia (a, d), Azerbaijan (b, e) and Armenia (c, f). Abscissa, days post vaccination (DPV); ordinate, percentage of animals with log_{10} VNT titres crossing the positivity threshold (>1.5). Empty bars, single vaccinated animals (LR-01 and LR-02 before booster); filled bars, double vaccinated animals (LR-02 group after booster). #, the positivity percentage was not indicated because the group is too small (1 or 2 animals).

Supplementary Figure S3. Percentage of Large Ruminants (LR) immunoreactive against FMDV serotype Asia1. Graphs showing the percentage of LR sera with positive VNT titres ($log_{10} \geq 1.5$) against FMDV strain Asia1/ASIA/Sindh08/TUR/15. Sera from Georgia (a), Azerbaijan (b) and Armenia (c). Abscissa, days post vaccination (DPV); ordinate, percentage of animals with log_{10} VNT titres crossing the positivity threshold (>1.5). Empty bars, single vaccinated animals (LR-01 and LR-02 before booster); filled bars, double vaccinated animals (LR-02 group after booster). #, the positivity percentage was not indicated because the group is too small (1 or 2 animals).

Supplementary Figure S4. Percentage of Small Ruminants (SR) immunoreactive against FMDV serotype O. Graphs showing the percentage of SR sera with positive VNT titres ($log_{10} \geq 1.5$) against FMDV strain O/ME-SA/PanAsia-2/TUR/07. Sera from Georgia (a) and Azerbaijan (b). Abscissa, days post vaccination (DPV); ordinate, percentage of animals with log_{10} VNT titres crossing the positivity threshold (>1.5). Empty bars, single vaccinated animals (SR-01 and SR-02 before booster); filled bars, double vaccinated animals (SR-02 group after booster).

Supplementary Figure S5. Percentage of Small Ruminants (SR) immunoreactive against FMDV serotype A. Graphs showing the percentage of SR sera with positive VNT titres ($log_{10} \geq 1.5$) against FMDV strain A/ASIA/Iran-05/TUR/06 (a, b) and FMDV strain A/ASIA/G-VII/NEP/84 (c, d). Sera from Georgia (a, c) and Azerbaijan (b, d). Abscissa, days post vaccination (DPV); ordinate, percentage of animals with log_{10} VNT titres crossing the positivity threshold (>1.5). Empty bars, single vaccinated animals (SR-01 and SR-02 before booster); filled bars, double vaccinated animals (SR-02 group after booster).

Supplementary Figure S6. Percentage of Small Ruminants (SR) immunoreactive against FMDV serotype Asia1. Graphs showing the percentage of SR sera with positive VNT titres ($log_{10} \geq 1.5$) against FMDV strain Asia1/ASIA/Sindh8/TUR/15. Sera from Georgia (a) and Azerbaijan (b). Abscissa, days post vaccination (DPV); ordinate, percentage of animals with log_{10} VNT titres crossing the positivity threshold (>1.5). Empty bars, single vaccinated animals (SR-01 and SR-02 before booster); filled bars, double vaccinated animals (SR-02 group after booster).
