# Supplementary figures and images for: Evaluation of Two Vaccines against Foot-and-Mouth Disease Used in Transcaucasian Countries by Small-Scale Immunogenicity Studies Conducted in Georgia, Azerbaijan and Armenia
Source: Vaccines (Basel). 2024 Mar 12;12(3):295. doi: 10.3390/vaccines12030295 (PMC10975580; doi:10.3390/vaccines12030295)

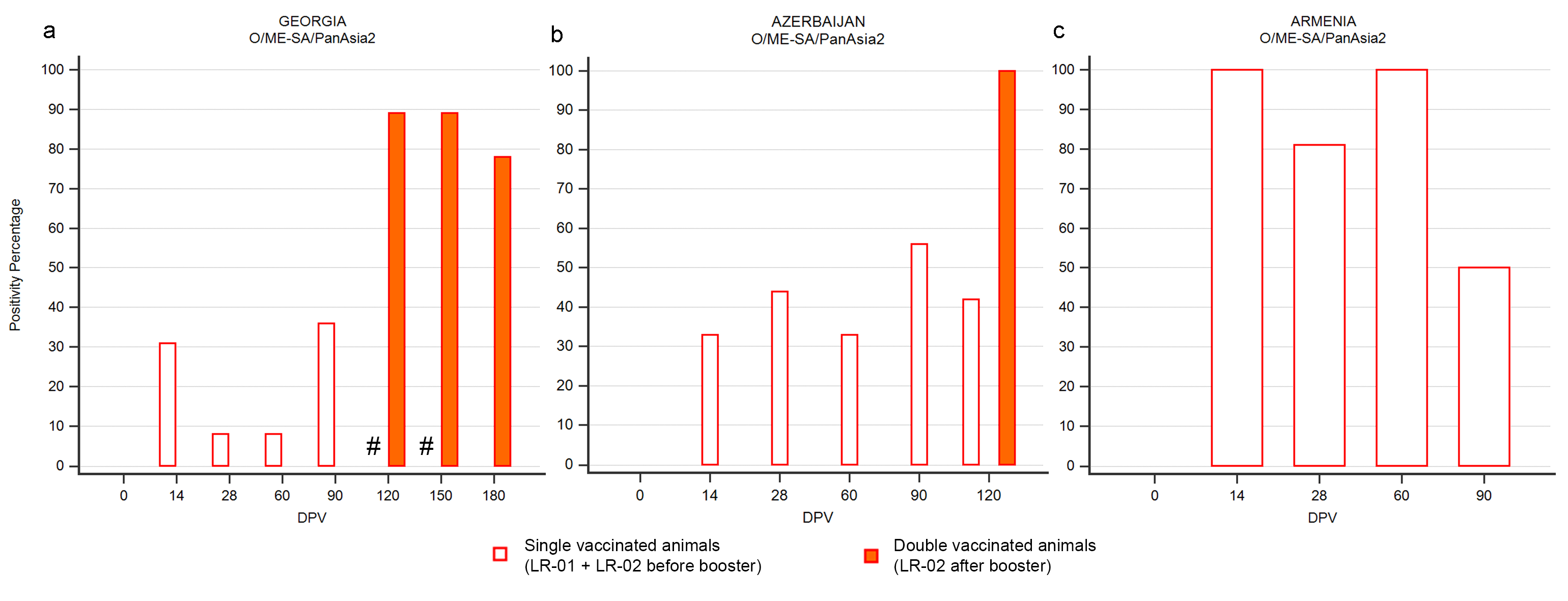

Supplement: Supplementary file 1 [file vaccines-12-00295-s001.zip › Supplementary Figure S1.tif]

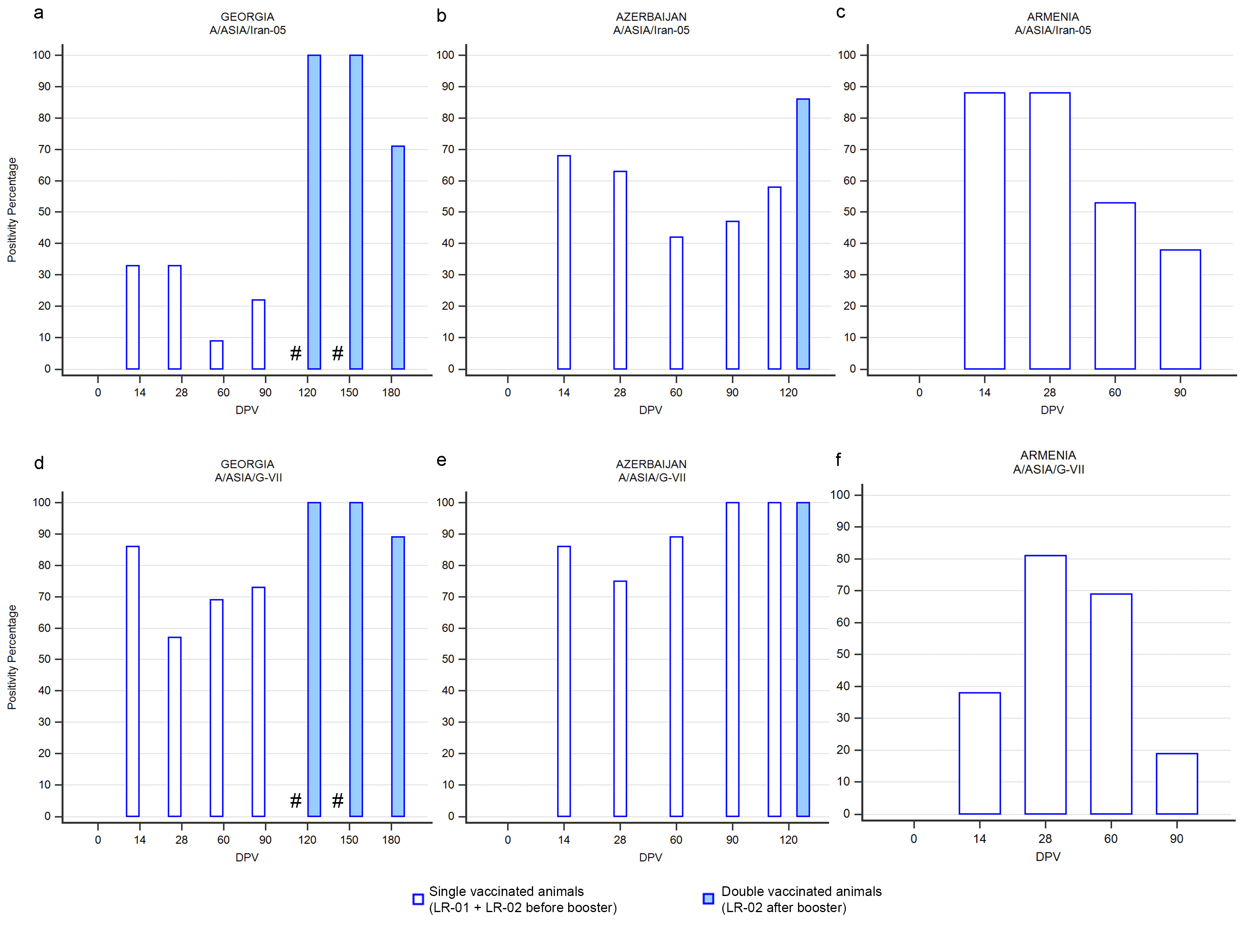

Supplement: Supplementary file 1 [file vaccines-12-00295-s001.zip › Supplementary Figure S2.tif]

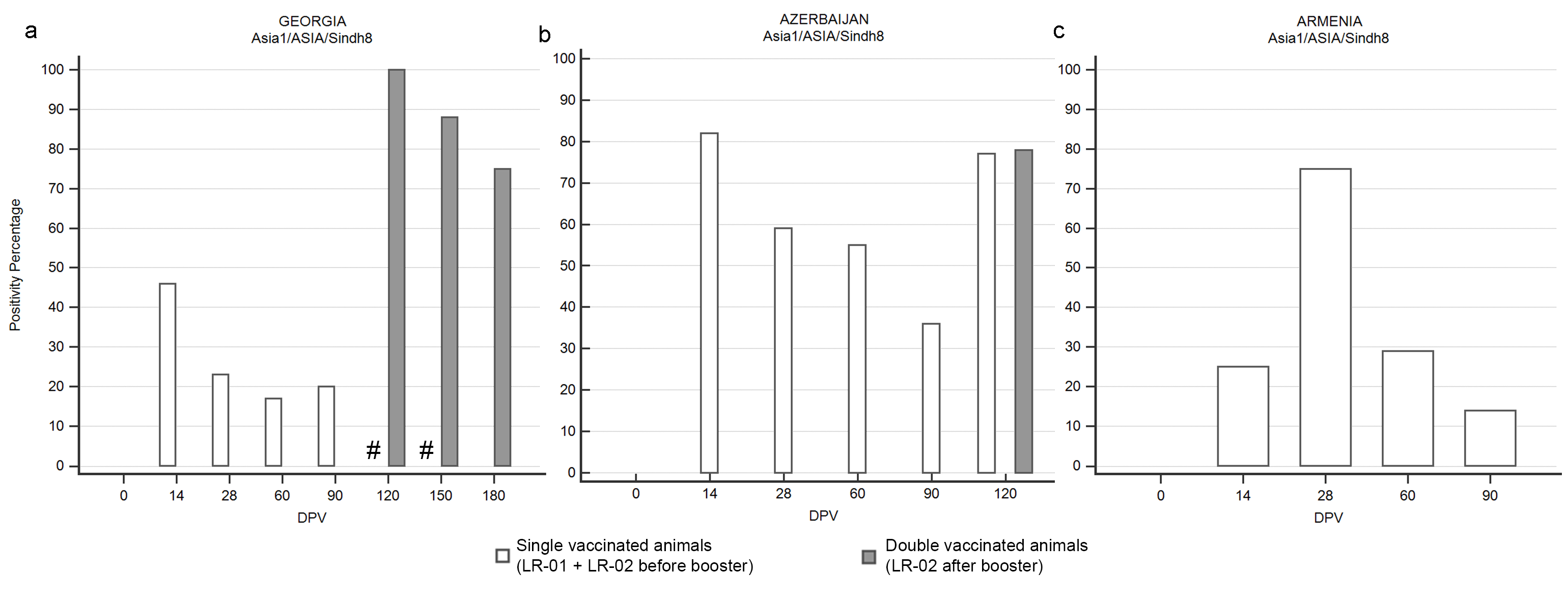

Supplement: Supplementary file 1 [file vaccines-12-00295-s001.zip › Supplementary Figure S3.tif]

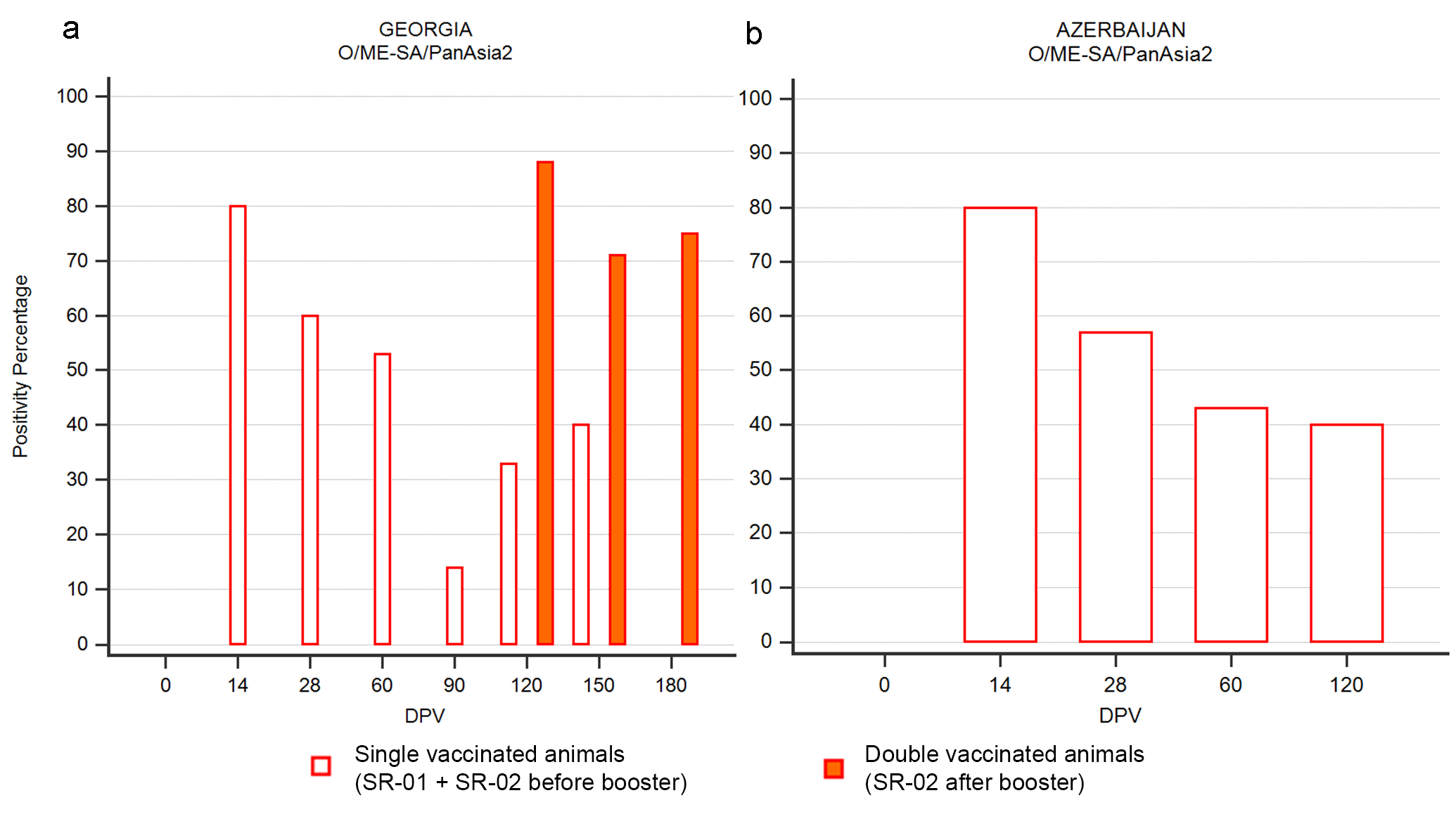

Supplement: Supplementary file 1 [file vaccines-12-00295-s001.zip › Supplementary Figure S4.tif]

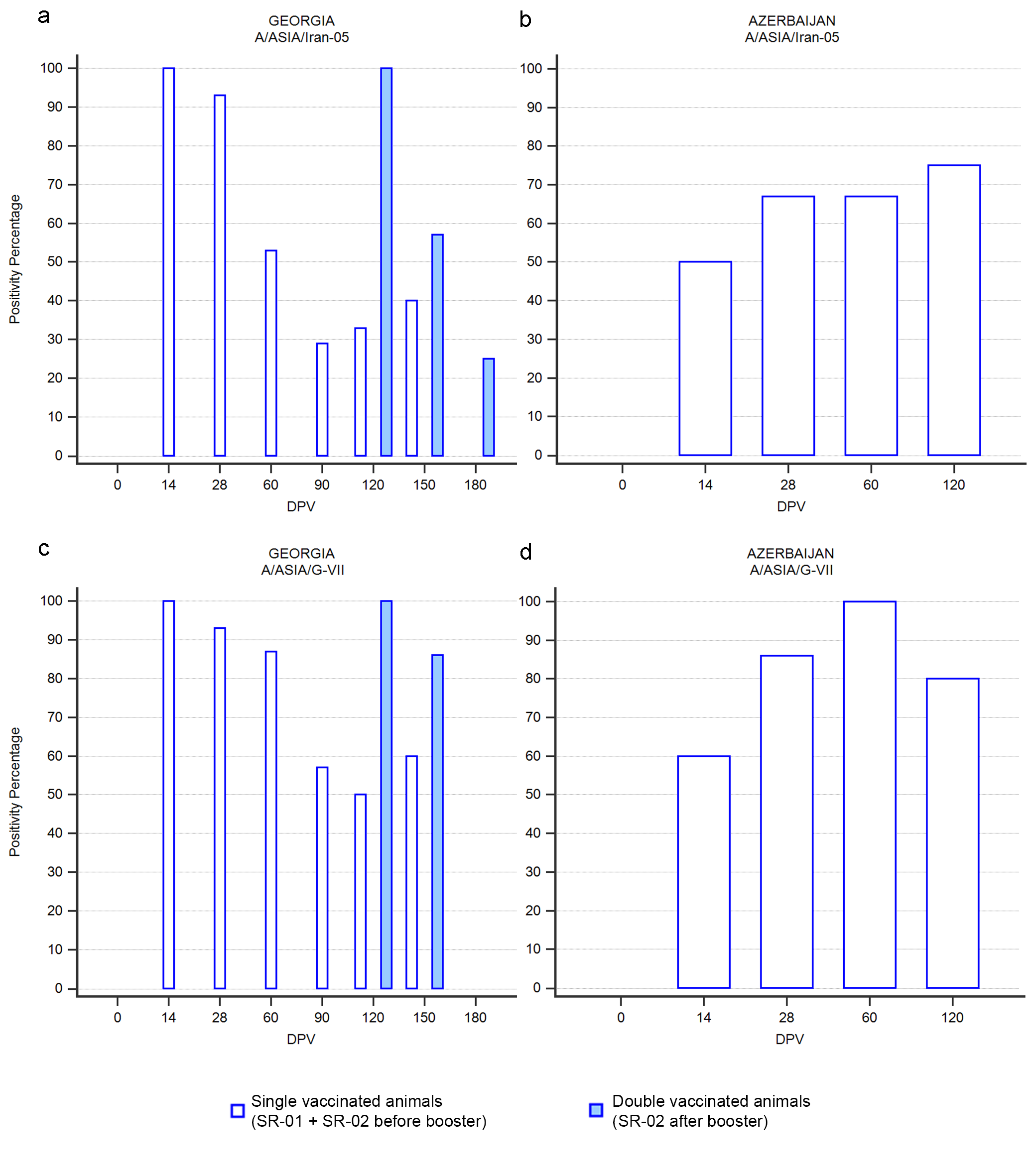

Supplement: Supplementary file 1 [file vaccines-12-00295-s001.zip › Supplementary Figure S5.tif]

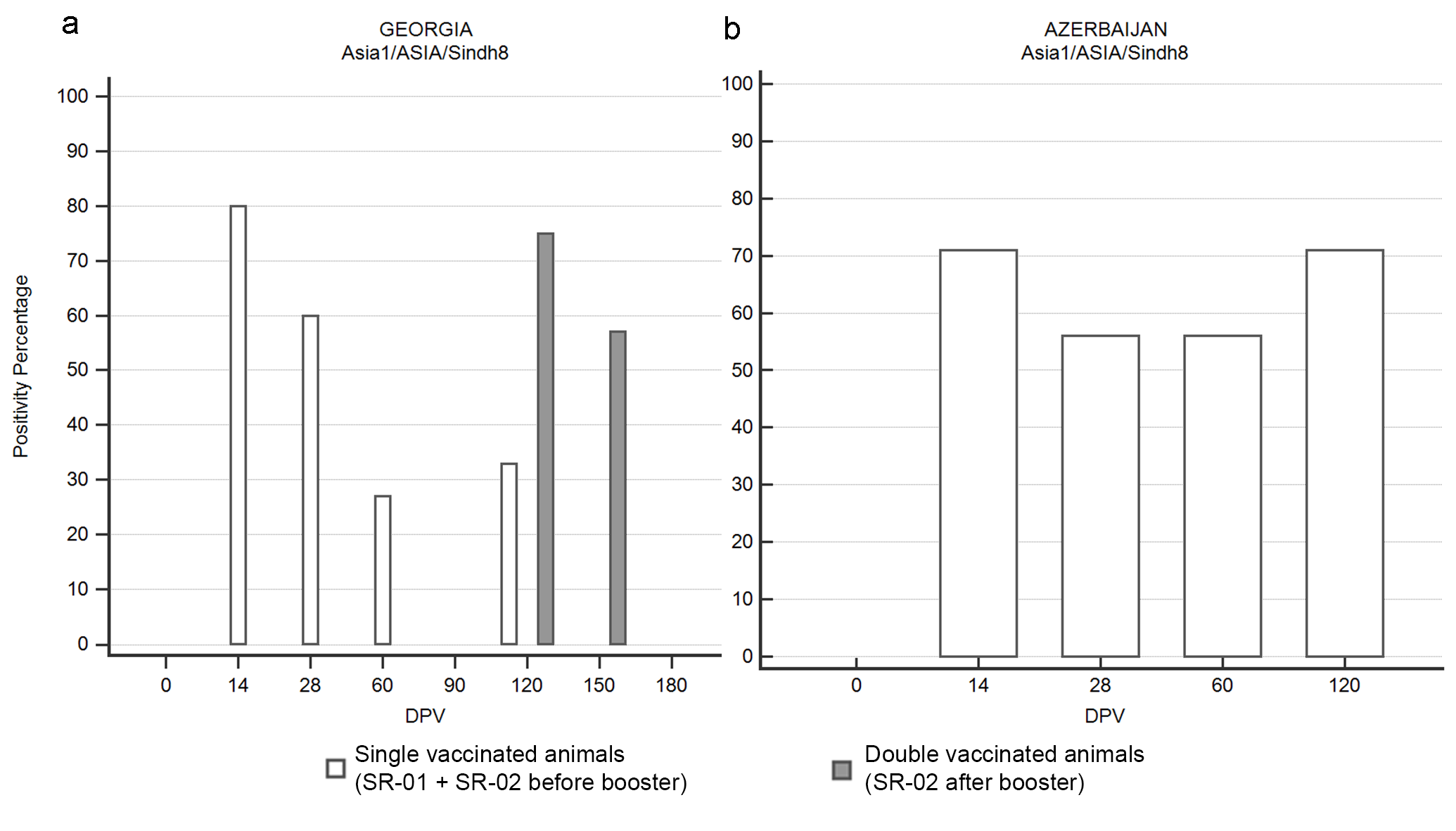

Supplement: Supplementary file 1 [file vaccines-12-00295-s001.zip › Supplementary Figure S6.tif]
